# A qualitative study of the background and in-hospital medicolegal response to female burn injuries in India

**DOI:** 10.1186/s12905-014-0142-5

**Published:** 2014-11-30

**Authors:** Nayreen Daruwalla, Jyoti Belur, Meena Kumar, Vinay Tiwari, Sujata Sarabahi, Nick Tilley, David Osrin

**Affiliations:** Prevention of Violence against Women and Children, SNEHA (Society for Nutrition, Education and Health Action), Urban Health Centre, 60 Feet Road, Dharavi Mumbai, 400017 India; UCL Department of Security and Crime Science, 35 Tavistock Square, London, WC1H 9EZ UK; Department of Surgery, Lokmanya Tilak Municipal General Hospital and Medical College, Dr. Babasaheb Ambedkar Road, Sion (West), Mumbai, 400022 India; Department of Burns and Plastic Surgery, Vardhman Mahavir Medical College and Safdarjung Hospital, New Delhi, 110029 India; UCL Institute for Global Health, Institute of Child Health, 30 Guilford Street, London, WC1N 1EH UK

**Keywords:** India, Poverty areas, Burns, Violence against women

## Abstract

**Background:**

Most burns happen in low- and middle-income countries. In India, deaths related to burns are more common in women than in men and occur against a complex background in which the cause – accidental or non-accidental, suicidal or homicidal – is often unclear. Our study aimed to understand the antecedents to burns and the problem of ascribing cause, the sequence of medicolegal events after a woman was admitted to hospital, and potential opportunities for improvement.

**Methods:**

We conducted semi-structured interviews with 33 women admitted to two major burns units, their families, and 26 key informant doctors, nurses, and police officers. We used framework analysis to examine the context in which burns occurred and the sequence of medicolegal action after admission to hospital.

**Results:**

Interviewees described accidents, attempted suicide, and attempted homicide. Distinguishing between these was difficult because the underlying combination of poverty and cultural precedent was common to all and action was contingent on potentially conflicting narratives. Space constraint, problems with cooking equipment, and inflammable clothing increased the risk of accidental burns, but coexisted with household conflict, gender-based violence, and alcohol use. Most burns were initially ascribed to accidents. Clinicians adhered to medicolegal procedures, the police carried out their investigative requirements relatively rapidly, but both groups felt vulnerable in the face of the legal process. Women’s understandable reticence to describe burns as non-accidental, the contested nature of statements, their perceived history of changeability, the limited quality and validity of forensic evidence, and the requirement for resilience on the part of clients underlay a general pessimism.

**Conclusions:**

The similarities between accident and intention cluster so tightly as to make them challenging to distinguish, especially given women’s understandable reticence to describe burns as non-accidental. The contested status of forensic evidence and a reliance on testimony means that only a minority of cases lead to conviction. The emphasis should be on improving documentation, communication between service providers, and public understanding of the risks of burns.

## Background

Most burns (90%), and most deaths as a result of them (98%), occur in low- and middle-income countries [1-3]. India contributes between 163 000 and 200 000 annual deaths to the global toll [2,4]. The epidemiology of mortality after burns in India is unusual in that deaths are more common in women than in men [4-14], particularly among the young adults who make up the largest group of admissions [12,14-16]. The female-to-male ratio varies over time and place, but lies in most reports between 1.1 and 1.6 [12,14,15,17-19].

Burns are usually classified by their agent - flame, electricity, or chemical – and may be accidental or non-accidental. Non-accidental burns may result from deliberate self-harm (attempted suicide) or from the actions of others (attempted homicide). Published figures put accidental burns at 41–95% of admissions (lower for women than for men), suicidal burns at 2–44% (higher for women than for men), and homicidal burns at 2–26% (also higher for women) [12,16-24]. India leads the world in intentional self-harm by burning [25], particularly in young women [6,14,15,21,23,26-28], self-immolation being elsewhere more characteristic of middle-aged men [3,29]. A recent analysis of the Million Death Study estimated 187 000 suicides in 2010, of whom 72 000 were women [30]. Of about 20 000 annual murders, 10–15% are of women, in whom burns are a common cause [31]. Again, the sex ratio of victims is the opposite of that seen in high-income countries, in which men predominate [25].

These statistics mask considerable uncertainty. Non-accidental deaths of young women – *dowry deaths*, *bride burning* – have long been a concern. They are considered most likely to occur within seven years of marriage and may be overtly homicidal or the result of suicide as a consequence of coercion or desperation. Attempt to commit suicide remains a crime (although decriminalization may follow a recent Supreme Court directive) [32]. The suicide of a young woman within seven years of marriage is considered suspect and her husband or in-laws may be held accountable. Similar recourse applies to suspected homicide.

Deciding on the cause of burns means choosing between three narratives. The first is that accidents are common and related to poverty, the safety of cooking appliances, and the use of kerosene or liquefied petroleum gas (LPG). The second is that deliberate self-harm by burning is a relatively common event with an established place in culture, from mythological to popular. The third is that immolation is a well-known method of homicide [33]. The ubiquity of kerosene means that it is implicated in many burns (between 33% and 77% in case series) [2,6,12,16,20,23,24,28,34-36]. As well as being a hazard, it is the vehicle used in over 95% of women’s suicides by burning [12,15], and is the usual accelerant in homicidal burns.

We wanted to hear the stories of women who had sustained serious burns, and of professionals who worked with them. We hypothesized that a woman’s decision to disclose the cause of her burns was the result of an interplay of competing pressures. Our study had three objectives. The first was to understand the problem of ascribing cause, by examining the human background to – and fallibility of – the recorded epidemiology of serious burns. The second was to understand the sequence of events after a woman was admitted to hospital, and how the actions of doctors, nurses, and the police might affect subsequent legal processes. The third was to understand opportunities for intervention to prevent burns and, failing that, to deliver justice.

## Methods

### Design and setting

The study had two components: semi-structured interviews with women (and their family members) admitted with flame burns to the largest units in Delhi and Mumbai over three months in 2012, and key informant interviews with doctors, nurses, and police officers who worked with burns cases. We wanted to work in more than one place in order to begin to understand differences between settings, but such a study had not been done before and we had concerns about its achievability. We chose Mumbai and Delhi as examples of metropolitan areas with large burns units that might be representative - at least to some degree - of different areas of India, and because of our existing research bases. We also wanted to ensure that support would be available for both interviewees and interviewers. We chose the burns unit in Mumbai because of existing relationships. ND and DO work with SNEHA (Society for Nutrition, Education and Health Action), a Mumbai-based nongovernment organization that provides counseling and crisis intervention services for survivors of violence. A multidisciplinary team assists women through psychosocial support, police consultation, and legal processes. SNEHA collaborates with the Mumbai hospital to discuss and support referrals for survivors of violence, run a women’s outpatient clinic, and provide counseling services in the burns unit directed by MK. We chose Delhi because JB had existing relationships with its police force. We discussed the study with directors of burns units at two hospitals, and chose Safdarjung Hospital because of the interest of VT and SS.

### Data collection

Three female investigators (KH, JM and RD) with postgraduate social science education and experience of qualitative research conducted interviews with women. Each attended a burns unit daily, became familiar with procedures and staff, and identified women admitted. In most cases, investigators were able to interview the client herself. When the client was unable to talk, investigators interviewed family and friends. Interviewers explained the purpose and content of the study, provided a participant information sheet, and received signed consent (or witnessed digit print where signature was not possible). Interviewers kept field notes describing their observations of practices on the ward, visits by police, doctors, and social workers, and their reflections on individual cases.

Two investigators (ND and DO) conducted semi-structured key informant interviews with doctors and nurses, and one (JB) interviewed police officers. Interviews lasted about an hour. They were conducted in private spaces after signed consent (oral consent by police officers). Interviews were audio-recorded, transcribed in full, and the transcripts pseudonymized. Data were stored in password-protected files. Transcripts were not returned to interviewees unless requested.

### Content of interviews

Interviews with women and their families were semi-structured and based on a topic guide. They began with questions describing age, education, faith, employment, marital status, sex and numbers of children, family composition, and accommodation. Subsequent topics covered the burn incident (when, where, who was there, what happened, cooking arrangements, clothing, other people injured), immediate action (sequence of events, who helped, what they did, where the woman was taken), immediate hospital care (experience on arrival, referral to another hospital, information provided, who accompanied the woman, necessary paperwork), care on the burns unit (sequence of events, who had talked to the woman, visits by social workers, police, or counselors, discussions with nurses and doctors), previous incidents and abuse (physical, emotional, economic, or sexual abuse, frequency, family conflict, injuries and effects on the woman, whether she had registered a complaint), and medicolegal action (what the woman would prefer, who might help, whether she wanted the police to be involved, what she would have liked them to do, what other help she needed).

Topic guides for key informant interviews covered professional background and experience with burns (numbers, sex differences, relationship with violence, homicide, suicide, accident, differences over time in the profiles of admissions, effects of the law), the sequence of events when a woman was admitted (what was done and who did what, communication between hospital departments, legal aspects of documentation, what nurses and doctors asked the client or her family, who handled medicolegal aspects of the case, guidelines or protocols, connections with social workers, visits by the police, changes of statement and dying declarations, interactions with lawyers and courts).

Topic guides for interviews with police covered professional background and experience of investigating burns cases (numbers, causes, trends, relationship with violence, homicide, suicide, accidents, common causes, sex differences), how first intimation happened, relationships with hospitals and liaison officers, magistrates, formats for filing First Information Reports, changes of statement, dying declarations and inquests, roles of levels of police officer, visits to burn scenes and collection of evidence, experience of prosecution).

### Interviewees

We developed case studies for 33 women: 13 in Delhi and 20 in Mumbai. Table 1 provides a demographic breakdown. Investigators considered admissions sequentially, but their ability to conduct an interview was subject to multiple constraints. Women were profoundly ill, some died, and contact was limited by the need for infection control, clinical activities, and visiting hours. Potential interviewees were therefore suggested after discussions with MK, VT, SS, and doctors and nurses on the wards. We interviewed 26 key informants - 12 clinicians and 14 police officers. The clinicians included burns nurses (3), residents (4) and consultants (2) working in burns, surgery and casualty departments, forensic pathologists (2), and a burns ward aide. Because SNEHA counselors had considerable experience of work with women in the Mumbai burns unit, we took the opportunity to interview three of them. ND and DO knew them well and they knew each other, and interviewing them together seemed a reasonable approach. Eight police officers were interviewed in Mumbai and six in Delhi (three Assistant Commissioners and 11 Inspectors).

**Table 1:** **Sociodemographic and burn characteristics of 33 women admitted to burns units at two urban tertiary hospitals**

**Age**	**n**	**Marriage**	**n**	**Accommodation**	**n**
<20 y	5	Unmarried	3	Chawl	9
20–24 y	16	Married	29	Flat	6
25–29 y	3	Separated	1	House	10
> = 30 y	9	**Marriage arrangement**		Room	2
**Literacy**		Arranged marriage	23	Zopadpatti (informal settlement)	4
Unable to read	12	Love marriage	5	Unknown	2
		Unknown	1		
Reads with difficulty	5	**Marriage duration**		**Site of burn**	
Reads with ease	13	<7 y	16	Kitchen	22
Unknown	3	7–10 y	5	Other indoor	1
**Schooling**		10–19 y	3	Unlocated	10
None	10	> = 20 y	4	**Burn type**	
Primary	5	Unknown	1	Flame	17
Secondary	11	**Children if married**		Kerosene	10
Higher	4	No	12	Unspecified	6
Unknown	3	Yes	17	**Total body surface area**	
**Religion**		**Male child if married**	12	<20%	1
Hindu	26	**Family structure**		20–29%	2
Muslim	7	Nuclear	22	30–39%	4
**Employment**		Joint or extended	9	40–49%	7
Never employed	15	Natal	1	50–59%	3
Not currently employed	6	Unknown	1	60–69%	3
Makes products	3	**Home ownership**		70–79%	4
Unskilled job	3	Client	1	80–89%	3
Shop, market, hotel, transport	1	Husband	1	90–100%	3
Student	1	In-laws	9	Unknown	3
Junior white collar	1	Natal family	3		
Schoolteacher	1	Quarters	1		
Unknown	2	Rental	16		
		Unknown	2		

### Analysis

We used framework analysis to understand accounts of burns and the sequence of events after admission. We chose this approach because we had specific objectives, a set of *a priori* questions, and a pre-designed sample [37,38]. Interviews were translated where necessary, transcribed, and read in full by three investigators. We indexed the data using textual codes, charted them by case, and mapped concepts and explanations [39].

The data reflected the accounts of participants and included both pre-specified and emergent concepts [37,40-42]. The framework was developed in MS Excel (Microsoft Corporation). ND, DO, and JB did the initial coding such that each transcript was coded by two researchers. In keeping with the structured sequence of topics, most of the categories were predefined: interviewee background, account of the burn event, who was present and what they did, where the woman was taken and how, what happened – in order – at the hospital, referral, visits by the police, social workers, doctors and nurses, initial and subsequent statements, case registration and subsequent legal action, antecedents and abuse, family relationships and household constraints. Key informant frameworks followed the topic guide in the same way.

Because of the structured nature of the data and the manageability of the numbers, we did not produce summary matrices, but included complete quotes in each matrix cell. One of ND, DO, and JB drafted summaries under each category within the framework, and it was discussed and edited by the others. Category grouping under themes was generally clear-cut given the sequence. Emergent themes were discussed after reading transcripts, matrices, field notes, and summaries.

### Ethics statement

The project was approved by the Institutional Ethics Committee of Lokmanya Tilak Municipal Medical College and General Hospital (IEC/14/12), Mumbai, the Ethical Committee of VM Medical College and Safdarjang Hospital (58-11-EC4/8), Delhi, and the UCL Research Ethics Committee (3546/002). It was overseen by SNEHA. Interviewers were trained to provide initial counseling and interviewees who reported violence were offered support from local organizations connected through networks working to end gender-based violence. Women and their families were provided with legal information and were assisted in filing cases after pre-litigation counseling and agreement. We also communicated with shelters and government organizations for further referral. Researchers were encouraged to debrief after each interview, and situations requiring intervention were discussed with the wider team to identify the best options for intervention and support for interviewees and their families.

### Role of the funding source

The sponsor had no role in the study design, data collection, analysis, interpretation or writing of the article. DO had access to all study data and responsibility for the decision to submit for publication.

## Results

From June to August 2012, there were 197 adult female admissions to the Delhi burns unit. The classification breakdown was 146 accidental (74%), 35 suicidal (18%), 14 homicidal (7%), and 2 unstated (1%). There were 86 adult female admissions to the Mumbai burns unit: 78 accidental (91%), 8 suicidal (9%), and none homicidal.

### Causes of burns described by interviewees and key informants

For the 33 women interviewed, 22 events were described by themselves or their relatives as accidental, five as suicidal, and six as homicidal. Key informants were unanimous in thinking that many burns were intentional, even when described as accidental: “… Burns – maybe homicide, suicide or accident – are more pronounced within the first ten years of marriage … where there is a hostile environment from the in-laws’ house and this young female is new to that house” (clinician). All key informants but one said that homicidal burns had become uncommon. Dowry death - the murder of a woman because of conflict over her natal family’s financial contribution to the marital family, particularly as a result of burning - is a familiar cultural trope. Police officers thought that it had become infrequent. “… Some of these cases that we see on TV and all that - the mother-in-law and father-in-law are burning her … It happens like that very rarely” (police officer).

There was a feeling that suicide was taking the place of homicide and that self-immolation was contributing to a greater proportion of burns. “I say homicides are few because no one holds them and sets them on fire: that is what I think. The second thing is suicide – all of them are suicides” (police officer). The area between homicide and suicide was hazy - “Dowry deaths due to harassment, it could be just harassment, to harass the lady to such an extent that she is forced to kill herself” (clinician) - and some interviewees saw contemporary dowry death as something more akin to systematic gender-based violence: “… Mainly because the husband gives trouble, her mental balance gets disturbed and she is fed up of life and she burns herself … We see more examples of dowry deaths of this kind” (police officer).

In some cases attempted suicide was private. In others there was a statement of intent, a performative threat in the form of an episode of self harm [43]. Several involved arguments between husbands and wives, as a result of which women openly doused themselves with kerosene and lit a match. Some women described their actions as an effort to make their husbands change their ways by provoking a sense of responsibility. “I thought that if I put kerosene on my hand, he would say he would not behave like this again … gambling, drinking” (burns survivor). In response to repeated beatings by a husband who drank heavily, one woman had doused herself three times. Her daughter said, “… this had happened before as well, so I didn’t pay attention to it. This was an everyday thing so I didn’t take on too much tension.” Participants and key informants suggested that young women had little idea of the consequences of burns. Likewise, familiarity with the scenario and the availability of kerosene meant that in two cases a husband told his wife to set herself on fire and, when she refused, lit the match himself (a drunken suitor did a similar thing and accidentally set himself alight). The reality of setting a woman on fire and the rapidity of spread usually unnerved the aggressor, who then helped to put it out.

### Space constraint

Space limitations were felt to be important: “… This is a slum area … The rooms are small, there is no separate kitchen. In that one room only, one sleeps, does everything … Then, the stove is sometimes kept down [on the floor], so from that there is a greater proportion of accidental burns here” (police officer). Counter-intuitively, intentional burns might be less likely in dense, urban informal settlements because “… Houses are set very close to each other and it is not allowed for anyone to do that. Because, for one thing, there are other people around and neighbours will also not allow this. If something is happening in the next house then, if that catches fire, then your house can also catch fire” (police officer); the assumption was that most burns were non-accidental and that social pressure prevented families from setting fire to women or abetting suicide.

### Problems with cooking equipment and fuel

Exposure to risk from stoves and their connections was common. Unblocking kerosene stoves with pins led to jets of kerosene ignited by the lit matches in women’s hands. Stoves that were ‘unstable’ or required shaking or salting were not given as much attention as usual. Pipes parted from gas-flow regulators bought from local dealers or were gnawed through by rats. Floor-level stoves flared up, kerosene stoves exploded, LPG stoves were left on or simply flared up. Candles or lanterns lit during nocturnal electricity outages set fire to clothing or other materials. Kerosene was both a risk and a weapon. It was the accelerant in all five reported instances of self-harm and all six reported attempted murders.

The grey market was a particular issue. “There are standard LPG cylinders which are issued by the government oil companies … The ones that cause the leakage are the smaller 2 kg cylinders which are filled … by local hawkers. … The regulators, the pipes, all these are available of cheap quality in the market … Because getting a gas connection is not that easy unless you have proper documentation” (clinician). Families often had a ‘government’ LPG supply, but the pathway from source to usage was complex. “Actually, we have one connection. With one connection we’ve got one stove and two cylinders … But it is at mother’s place. What we have is the same cylinder … they bring it to the street outside … they transfer gas from big cylinders to smaller ones … the stove that we have is local” (survivor’s father).

### Inflammable clothing

In 17 cases, specific references were made to women’s clothes catching fire. Spread – often unnoticed at first – was possible when a woman was wearing a scarf (*dupatta*), a *sari* (the *pallu* of which hung behind her), or a synthetic *maxi*, a loose-fitting nightgown often worn at home. “My *dupatta* was hanging and caught fire, slowly, slowly, burning more and more, while I was paying attention to the milk … we throw it behind us and then we don’t pay attention to it” (burns survivor). Accidents were often explained by lapses in attention for which women blamed themselves - “Then suddenly I didn’t put the pin of the stove in properly… “ (burns survivor) - or were blamed: “Actually, the pipe was leaking, and she used to put a polythene wrapper before putting the pipe on. It was not leaking, I mean it was loose. It used to come out with pressure. I had told her also … I had told her in front of my mother” (survivor’s husband).

### Antecedent household conflict, gender-based violence, and alcohol use

Quarrels, abuse, and alcohol dependency were often in the background, regardless of the immediate cause. Three attempted homicides were committed by husbands in the midst of violent arguments and three were described as following domestic violence and alcohol problems. “For the first 6–7 months things were fine between them. After that it’s been like this … He hits, he drinks and comes and hits …” (survivor’s brother). The longevity of abuse tended to numb responses to it. “For the last eight years he has been hitting me … I told my brother, told my mother. They kept saying, have patience, initially all men are not good. I kept patient and endured, and now he burnt me like this” (burns survivor). In a case of homicidal burns, seven years of sustained deprivation and physical abuse had led to the involvement of a local civil body, but violent events were so regular that the woman’s brother did not go and collect her when she called (survivor’s father).

Particularly pernicious was the combination of alcohol use, unemployment and domestic violence. “Every day he used to fight. The whole day he would not work, nothing … Today it’s been 17 years … My mother stayed because of us. Otherwise she would have gone back to her parents’ house a long time ago … He used to hit as well. Because he hit her, she tried to burn herself” (survivor’s daughter). The blurred distinction between homicide and suicide is illustrated here, as it was by the fact that several women admitted with accidental burns described histories of domestic abuse. Violence was often seen as normal: “… One vessel will naturally bang against another” (burns survivor). “There are very few men who don’t hit their wives” (burns survivor). Interviewees’ accounts frequently included a suggestion that the woman herself was implicated: “He used to beat me up at night, not in the daytime. He would switch on the cooler … nobody’s room is close by … If there was noise, mother-in-law would say, why? You give him trouble and that’s why he beats you up” (burns survivor).

### Initial care-seeking after the incident

Accompanied by their husbands, family members or neighbours, it took women between half-an-hour and three hours to reach hospital. Initial care-seeking suggested a preference for private hospitals (23 of 33 women), partly because of their proximity and reputation, but also because of concerns that government hospitals would be more likely to register complaints. Fear of the law was general. When a woman’s natal family asked why her in-laws had not taken her to hospital for three hours, they said “… you would say we burnt her (survivor’s mother).” The possibility that a history of abuse would trigger retribution made the decision to seek care complicated: “Her husband beats her and does this and that … So they were saying that this will become a police case … and by then my mother’s life would have gone. So that’s why we did not admit her there” (survivor’s daughter).

Women seen at peripheral hospitals were usually transferred to a public tertiary hospital within 24 hours. Some families said that they were prompted to transfer women because they thought that the hospital was unhygienic. In other cases, private hospitals lacked the expertise and facilities to treat burns, or seemed worried that their reputation might be damaged if the woman died (survivor’s father).

### Clinical and medicolegal sequence of events

**Figure 1:**
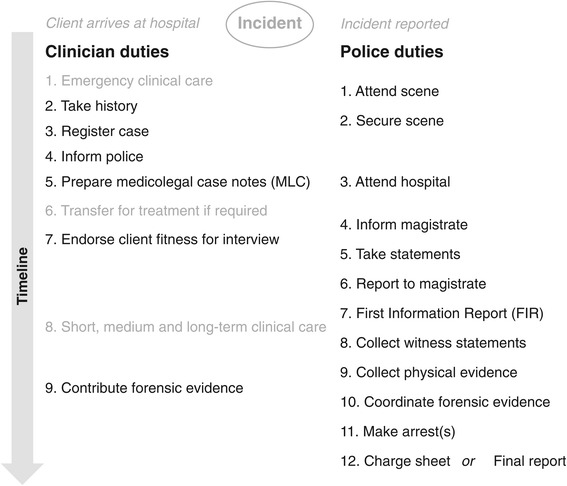
**Schematic summary of duties of clinicians and police when women are admitted to hospital with burns.** Steps beginning with the lodging of a First Information Report depend on an allegation being made.

Figure 1 summarizes the processes that clinicians and police had to work through in responding to burns admissions. The stages of action were relatively clear from clinicians’ accounts: immediate clinical care, registration, admission to hospital, informing the police, clinical note-taking and medicolegal documentation, forensic pathology, and – later - court proceedings, if initiated. The processes by which these were achieved differed in detail in the two units.

Despite some client interviews suggesting that doctors were occupied with medicolegal formalities, they all said that a rapid clinical response was their priority. “Your duty as a doctor would be to save the life of the patient first, and there are various Supreme Court judgments supporting this … Thereafter, if you get some time, you do a police information” (clinician). Experience meant that both burns teams had developed relatively smooth processes in which medicolegal paperwork and urgent clinical action ran in parallel. Medicolegal processes began immediately after clinical stabilization.

In India, any burn requires registration as a ‘medicolegal case’ (MLC). Each client was entered into an MLC register and given an identification number - the process linked explicitly with a requirement to inform the police. Clinicians had a clear – if self-protective – understanding of the implications of documentation. ”Why an MLC? Simply because tomorrow … the MLC is a document which once has been made cannot be tampered with … so that makes you very safe … If, suppose, somebody alleges foul play … you have secured yourself because you have documented it, everything in black and white…” (clinician). Working in tertiary hospitals, clinicians often received clients transferred from other hospitals. Their experience of the legal process made them careful to revisit the documentation. Clients often arrived with MLCs, but they tended to file new ones, despite the effort of duplication. “It is safer to have an MLC in hand than to not have an MLC at all … We want to play safe because later on if there is no police case and the patient expires then it is tough” (clinician).

### Medicolegal roles of clinicians

Clinicians were concerned to carry out their duties thoroughly within their remit, but not to extend it. “We do not talk to the patient in terms of the actual history … because once it has been documented we do not wish to be seen as somebody trying to interject thoughts into the patient’s mind” (clinician). They saw themselves as vulnerable to accusations of partiality: “… I don’t want to be seen as somebody who has tried to influence the decision of the patient in terms of the history. My job is merely to document what I have got from the patient in the patient’s own words” (clinician). Clinicians were required to certify that clients were fit for interview by the police. They emphasised certification at the end of the interview because cross-examining lawyers might suggest that they had deteriorated during the statement.

### Roles of police and magistrates

Nominated police officers were present in both hospitals and communication was rapid. “In the casualty there is a police who is situated there … And he is informed as soon as admission is made … I don’t think he really has much choice. He has to make a police case” (clinician). The officer was responsible for completing police documentation on site and informing the appropriate police station. If the incident had occurred in the city, the officer informed the police station at the woman’s point of origin and officers were required to come to the hospital to take statements. If the incident had occurred at a distance – outside the city or state – local police officers were able to take statements in lieu of officers from the woman’s point of origin, but there were often delays in communication and in the decision about who would take responsibility. Hospital registration systems were in place and reminders were sent if the police had not attended.

Designated police officers visited the hospital – usually within hours of admission - to take more detailed statements, as well as visiting the site of the event. “Then immediately the IO [investigating officer] and SHO [station house officer] will go to the hospital. First he will try to get her statement … if she is in a position to give a statement then immediately SEM, Special Executive Magistrate, will be informed. He will come to the spot and record her statement” (police officer). The involvement of magistrates was thought to be important, even though there was some uncertainty about the legal requirement for it. “The SDM [Sub-Divisional Magistrate] comes … for all the … female patients who are burnt less than seven years. Whenever dowry is suspected, the SDM comes. Because whatever statement has been given to SDM, that I understand carries greater weight in the court of law” (clinician). Even more than the clinicians, the police feared accusations of lack of effort or partiality. “We can register, we don’t have to wait for the SDM, but we do so because we are the police: tomorrow no one should question our credentials, so it is better if they also come and it is done properly” (police officer).

### Initial statements

All the key informants – clinicians, police and social workers – said that the initial statement given by the client or accompanying people usually described an accident, and all assumed that this was usually not the truth. ”… Many of the patients come in the casualty and … they usually give you that it is because of a stove blast or because of some accidental fall of a *sari* over there and she got afire” (clinician). Interviewees voiced their scepticism - ”… I really don’t understand that with one candle how one can get burnt up to 90% to 100%” (nurse) - but sympathised with women who had suffered burns: “The patients say they are scared of the in-laws, the husbands, what treatment they will get later on when they go home … That’s why they never admit” (nurse). At the same time, they felt that it was inappropriate to examine clients’ accounts more deeply, particularly in the early stages of admission. “… Patient will be young, newly married, so we have some queries looking at it. It is not accidental, we feel it sometimes. But those patients, we cannot ask them directly … Because even they feel bad … So you just accept as it is” (nurse). Workload and the realities of facing a group of family members in an emergency made a matter-of-fact approach advisable: “We do not have that much time to take the history. And also taking a history in that much detail at that particular time is also not good, because the relatives all were standing and seeing your face: what you are going to do?” (clinician).

### Changes to statements

Over the course of their hospital stay, clients often changed their accounts of what had happened to them. Although the feeling was that such changes were more likely to reflect the truth, this was not always the case. Family pressure might lead to withdrawal of allegations: “… If they are made cautious then the accused is made cautious, then they will again change the mind of that female by pressure tactics” (clinician). Conversely, a common occurrence was that “… the in-laws bring the patient. In the casualty the patient is told by the in-laws that, ‘when they ask you for the statement just say that it is a stove or a gas burn.’ Then the patient’s relatives … come and after they arrive the lady changes her statement and states it as homicidal burn and says that she has been burnt for dowry” (nurse).

The procedures for documenting this were probably the least certain in the minds of interviewees, although it is possible that this reflected reticence to be involved. In general terms, clinicians felt that, ”… If the patient wishes to change the statement, then we have to inform the police in the hospital to contact the police from that police station, and then … someone from that station would come back” (clinician). This course of action was appropriate, but the standing in court of a documented statement might, in the opinion of the interviewees, vary. “… In case there is an allegation and … patient has changed the statement … more often than not … they find that it is more important that the SDM sort of validates what has been said” (clinician).

### Dying declarations

Sometimes the lack of clarity on documentation was heightened by the fact that the woman wanted to make a statement in extremis. This is known in India as a Dying Declaration (DD). In principle, “For the DD we always inform the SDM. We try to ensure that the statement is done in the presence of the SDM … If we feel that she is going to die very soon and she wants to tell us something and we do not have that much time, then the IO tries to record her statement in front of the doctor, or tries to get the doctor to record it” (police officer). Experienced clinicians said that the DD could be taken by an attending doctor, although few had done so. “These municipality doctors are so busy, they have such a heavy workload and so many patients are coming. This means they would have to keep going to the court if they record the Dying Declaration. So that is why they hesitate a little. If there is absolutely no other choice then they sometimes do record it. It is not as if they absolutely don’t, but it is rare” (police officer).

### First information report

Prepared by the police, a First Information Report (FIR) documents a cognizable offence: a crime that the police are empowered to investigate without applying for a court warrant. In principle, ”After taking a statement, immediately the investigating officer, considering the prima facie facts, draws down a FIR. Then they have to take a statement from the complainant, the relatives of the complainant, and appropriate section would be filed” (clinician). At the same time, “The police goes to their house, scrutinises the house and a *Panchnama* [first report on evidence] is done” (police officer). In principle, again, the FIR may result from a statement by a complainant. “… Sometimes the parents say that my daughter had some troubles earlier, she might have tried to burn herself as a result. If they say that, then we take the FIR from the parents or the brother” (police officer). The same interviewee added, “And if absolutely no one is there, then the police officer can lodge the FIR themselves,” a statement echoed by others: “If we see foul play, then it is our duty to register the case” (police officer). Key informant interviews suggested that this was an account of an ideal situation that often does not obtain. First, the process of securing an FIR usually required substantial efforts on the part of the complainant. Second, it was unusual for the police to lodge an FIR of their own volition (*suo motu*) in the absence of an allegation: “We will go by the statement of the victim or her relatives, blood relations, and not by the evidence” (police officer).

### Subsequent legal process

Table 2 summarizes the legal framework around burns, both fatal and non-fatal. Key informants said that the pursuit of justice through the courts turned almost solely on allegations and testimony from either the client or her family. Despite the activities of forensic pathologists, and barring obvious discrepancies between prosecution or defence accounts and the clinical evidence (for example, a degree and pattern of burns incommensurate with the story), “Circumstantial evidence is not decisive evidence. That may be supporting evidence, but you cannot use it to make any decisions” (police officer). This was true of both crime scene evidence collected by the police and forensic evidence collected by pathologists: “There have been various notifications since Supreme Court judgment that clinical evidence in this country is the poorest … So, the benefit of doubt as per the law is always given to the accused and there are reported cases where with this benefit of doubt the accused has been acquitted” (clinician).

**Table 2:** **Legal provision for women in India who suffer burns**

**Code of Criminal Procedure, 1973**	**Response by police and magistrate**
Section 174	Response to suicide, homicide, accident, or death under suspicious circumstances.
	Applied particularly to women within seven years of marriage.
	Duty to report incident to magistrate empowered to hold inquests.
	Duty to proceed to the place where the body of the deceased lies, and, in the presence of two or more residents of the neighborhood, make an investigation and draw up a report of the apparent cause of death, describing signs of injury and how they appear to have been inflicted.
Section 176	Duties of Magistrate empowered to hold an inquest.
	Amended in 2005 to apply to death in custody.
**Indian Penal Code, 1860**	**Charges**
Section 304	Punishment for culpable homicide not amounting to murder.
	If the act that led to death was done with the intention of causing death or injury likely to cause death, or with the knowledge that it was likely to do so but not the intention of causing death.
	Minimum sentence: fine.
	Maximum sentence: imprisonment for life or 10 years.
Section 304B	Punishment for dowry death.
	Death by burns, injury, or in other abnormal circumstances of a woman within seven years of marriage, if it is shown that soon before her death she was subjected to cruelty or harassment by her husband or his relative in connection with a demand for dowry.
	Minimum sentence: 7 years.
	Maximum sentence: imprisonment for life.
Section 306	Abetment of suicide.
	Minimum sentence: fine.
	Maximum sentence: imprisonment for 10 years.
Section 498A	Cruelty by husband or his relative.
	Willful conduct that could drive a woman to suicide, serious self-harm or ill-health, harassment with a view to the unlawful acquisition of property or valuable security, or harassment when she does not meet such demands.
	Cruelty defined as willful conduct of a nature likely to drive the woman to commit suicide or to cause grave injury or danger to life, limb or mental or physical health; or harassment with a view to coercing her or any person related to her to meet an unlawful demand for property or valuable security, or on account of a failure to meet such demand.
	Minimum sentence: fine.
	Maximum sentence: imprisonment for 3 years.

The time for cases to come to court had been decreasing. “For the court summons, earlier, 5–6 years back, it used to be about six years, eight years, ten years … but now there is some legal binding that they have to settle the cases within a certain stipulated period of time. So now the summons come early - within a year, within two years - and then it depends upon the patient’s ability to pay money to the lawyer, probably” (clinician). The court usually summoned the doctor whose name appeared on the MLC, or another clinician to report on the notes if the former was unavailable. The clinician’s responsibility was primarily to discuss the content of the medical records. ”We are medical people … We are not involved because we cannot become a party … We only state what we have seen and what we have found. Our role is limited to that” (clinician). A recurrent concern was the tendency of defence lawyers to cast doubt on the fitness of the woman to give a statement. “I am not well-versed with law … That is why the defence, from the husband or whatever, they try to find something weak in our statement. So we have to be ready for that … because lawyers are more knowledgeable about medical things than we doctors are about law” (clinician).

Given the need for sustained action by the complainant, and the limited utility of circumstantial evidence and *suo motu* action, low numbers of convictions were to be expected. “I have been going to court for so many years to give evidence. I know of only two verdicts that have … given justice to the woman who was burnt to death” (clinician). The pressures on women to report intentional burns as accidents did not go away. Indeed, women who had recovered were in many ways more vulnerable. “… The ladies are still … married … they have to go back to their house again. That’s why they don’t register any case against the husband, with the in-laws. They say, we have children, we have to go and face them again, and again they may trouble us” (nurse). In contradistinction to an image fostered by popular fiction, the police saw themselves as particularly vulnerable to accusations of partiality, the evidence they gathered was generally inadmissible, and the state rarely prosecuted of its own volition.

Some key informants implied that the laws designed to address violence against women were being used to take unjustified revenge on marital families. “This dowry act law is a very dangerous law … a large number of court judgments are coming, specially from different high courts, and there is rampant misuse of this anti-dowry law. Unknowingly or knowingly, alleging and getting hooked up all the relatives of the so-called husband. That is very detrimental to the society” (clinician).

## Discussion

### Summary of findings

Interviewees described accidents, attempted suicide, and attempted homicide. Distinguishing between these was difficult because the underlying nexus of poverty and cultural precedent was common and action was contingent on potentially conflicting narratives. Space constraint, problems with cooking equipment, and inflammable clothing increased the risk of accidental burns, but coexisted with household conflict, gender-based violence, and alcohol use. Most burns were initially ascribed to accidents. Changes of statement were possible, although their legal admissibility was often uncertain. Clinicians adhered to medicolegal procedures, the police carried out their investigative duties relatively rapidly, but both groups felt vulnerable in the face of the legal process. Women’s reticence to disclose non-accidental causes and the limited quality and validity of forensic evidence underlay a general pessimism.

### Overlapping risks: the firestarter nexus and the narrative precedent

**Figure 2:**
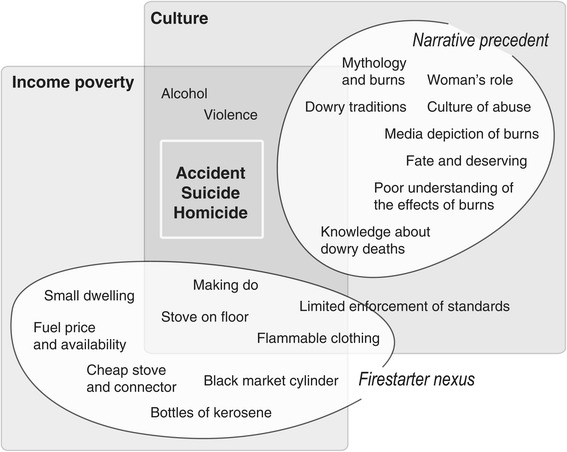
**Some ideas about the background to burns to women, with two overlapping themes.**

Our findings lead us to suggest two general contributors to the risk of burns to women in India, summarized in Figure 2: environmental risk and cultural precedent. Women are exposed to a cluster of potential hazards that make burns more likely, a combination of parsimony, environment and behaviour that we call the *firestarter nexus*. Economic constraint means smaller homes and a combination of frugality and tradition – making do – that includes wearing synthetics, cooking on the floor, accessing materials through the informal sector, and using kerosene. There is some evidence that burns are becoming less common, partly because improving socioeconomic conditions, changes in cooking fuel use, and increased safety have led to reductions in accidental burns [19].

While constraint is a determinant, it is not the whole story. That there is something in south Asian cultures that makes immolation of women more common is undeniable given the incidence of serious burns. This *narrative precedent* has been dwelt on substantially in the literature [31,44,45], with an emphasis on mythological tradition and patriarchy. Our interviewees talked more about the motif of burning in television soap operas – followed by recovery with no visible sequelae – which presumably echoes ancient images of purification, *sati*, and the trials of Sita in the *Ramayana*. The ‘stove blast’ is both a real hazard and a means of explaining away intentional burns that has become shorthand for describing incidents, often somewhat vaguely. Violence against women is widespread, and a scenario in which systematic abuse pushes women to suicide is sufficiently established to give it a section in the Indian Penal Code, yet little consideration in wider society.

### Risks associated with cooking and kerosene

Most burns to women in India are caused by kitchen flames [4,12,17-20,28,34-36,44], and their incidence peaks at cooking times in the morning and evening [15]. Although stove accidents remain the commonest explanation [4,14,20,24], deaths have often been suspected to be non-accidental. The peak age-group is women in their twenties [12,14,15,20,22,45], and burn patterns often do not match the descriptions of events.

In a study of eight Indian cities, kerosene was used as cooking fuel in 73–98% of poorer households [46]. Storing kerosene, the pouring process, the pumping necessary to pressurize the system, and the regular need to unblock it are all potentially hazardous [20]. LPG stoves usually run on cylinders supplied by government-accredited dealers. Proof of residence is required and there is a thriving market in decanted cylinders and substandard equipment. Cylinder explosions are unusual and tend to lead to multiple casualties, but gas leaks from insecure connections are probably more common and cluster with grey market provision [19,23,35]. The tendency for Indian women to wear loose-fitting garments and the popularity of silks and synthetics are also believed to be risks [4,12,18,20,24,26,34,35,45,47,48].

### The legal process

There is some evidence (albeit potentially reflecting changes in reporting) that murders of women have decreased and suicides have increased [21,30]. The legal framework is both stringent and well-known [31,44,45,49-52], and it is certainly possible that knowledge of the consequences has led to a fall in dowry-related homicide. However, the protean nature of abuse means that its scope extends beyond attempted murder after pecuniary conflict.

There was a dissonance between fear of the law and belief that it was unlikely to lead to punishment in an environment in which only one in three domestic violence cases results in conviction [53]. In deciding where to take women with potentially fatal burns, families factored in their concerns that they would be blamed, concerns that public hospitals would spend time on medicolegal procedures at the expense of lifesaving clinical interventions, and beliefs that care would be better at private hospitals. We might argue that these concerns illustrate a narrative of bureaucracy with substandard public services that extends beyond burns to daily life in contemporary India. Our findings, however, technically undermine the narrative. The clinical management of burns is an expensive long-term proposition and there is little doubt that they are best treated in specialised public sector units with concentrations of expertise and high client throughput. We saw no evidence that clinicians working in these units compromised clinical care in favour of investigation and paperwork. Our interviews were limited to the context of specialist units, and it is certainly possible that some of the clients’ fears might be justifiable in lower-tier institutions. A similar caveat applies to descriptions of the early medicolegal procedures. The hospitals registered and documented MLCs, the police were on hand and investigating officers attended rapidly, interviews were taken and evidence collected, and magistrates were informed appropriately.

### External validity and bias

The study is as subject to potential criticism as other qualitative studies that involve purposive sampling and are not designed to examine epidemiology. The two hospitals were tertiary centres and their case-mixes cannot be assumed to be similar to those of hospitals in other cities, towns and states, or of private hospitals. Nevertheless, we chose them because of their high volume of admissions and experienced staff, many of whom had worked elsewhere and had considerable medicolegal experience. Interviewees’ opinions may or may not have reflected the actual numbers of serious burns to women, or their causes. This is the point and we see no reason to doubt that the difficulty of knowing whether burns are accidental or non-accidental is a serious concern.

Two more potential biases merit consideration. First, because of the sensitive nature of the study and the realities of a burns unit, interviewees were selected after consideration by their care providers. This means that the sample was more likely to include survivors. We do not know whether it reflected a bias toward accidental burns (to avoid combative territory) or a bias toward homicidal or suicidal burns (because they were considered by others to be germane to the study). Second, interviewers were aware of the potential causes of burns and, like anyone else, tended to form an opinion on whether they might be non-accidental. The transcripts suggest that they preferred sensitive questioning to persistent probing, and for this we make no apologies.

### Recommendations

Some challenges were amenable to improvement. We recommend protocols for documenting MLCs that would make them thorough and robust in court (with, perhaps, an emphasis on peripheral institutions which clients attend first). One interviewee suggested external scrutiny of paperwork to make sure that it is satisfactory. We recommend new protocols that simplify the duties of police when clients come from a distance, and a clearer understanding and chain of responsibility for clinicians and police in recording Dying Declarations.

Several interviewees recommended public awareness of the dangers of burns and ways to reduce kitchen hazards. There is also a place for mass media involvement, through campaigns and storylines in popular broadcasts. In the medium term, steady changes in fuel use patterns should help, but we do not think that legislation on the use of kerosene is likely to be useful, particularly as it would be more likely to affect poorer families.

Other challenges to uncovering truth and delivering justice are more daunting, including a narrative of misuse of the law in which families wilfully implicate other families with accusations of homicide or abetted suicide. The reliance of the legal process on complaint and testimony combines with the relative lack of credence attributed to circumstantial evidence to make FIRs hard to register, charges hard to draw up, and cases hard to win [54].

## Conclusion

The *firestarter nexus* and the *narrative precedent* underlie accidental, suicidal, and homicidal burns. The similarities between accident and intention cluster so tightly as to make them challenging to distinguish, especially given women’s understandable reticence to describe burns as non-accidental. Added to this is the contested status of forensic evidence and a reliance on testimony. Police officers often lack motivation to distinguish causes in cases in which clients are initially reluctant to make an allegation, or when the circumstances of the case make it unlikely to result in conviction. The emphasis needs to be on improving record-keeping, communication between service providers, and public understanding of the risks of burns.
